# Blueberry Anthocyanins Ameliorate Hepatic Dysfunction in High-Fat Diet-Fed Mice: Association with Altered Gut Microbiota and Bile Acid Metabolism

**DOI:** 10.3390/foods14173121

**Published:** 2025-09-06

**Authors:** Peng Xu, Yucheng He, Junyi Wang, Yingkun Sheng, Jianfeng Wang

**Affiliations:** 1 College of Life Sciences, Zhejiang Normal University, Jinhua 321004, China; 18267163445@163.com (P.X.); zjnuwjy@163.com (J.W.); 2 Xingzhi College, Zhejiang Normal University, Jinhua 321004, China; hyc585058@163.com; 3 College of Life Sciences, Shanghai Normal University, Shanghai 200234, China

**Keywords:** obesity, blueberry, gut microbiota, bile acid

## Abstract

The rapid rise in obesity has evolved into a critical global public health concern. Considering the potential adverse effects of current anti-obesity medications, the development of functional foods sourced from natural materials has emerged as a viable alternative. Blueberries, a category of berry fruits, exhibit potential anti-obesity characteristics. In this research, we assessed the impacts of Blueberry extract rich in anthocyanins (BE) on lipid metabolism and liver health in a high-fat diet (HFD)-induced obese mouse model. The findings indicated that BE notably diminished lipid accumulation in both serum and the liver, and mitigated hepatic steatosis and oxidative stress. Integrated proteomic, metagenomic, and metabolomic analyses further revealed the underlying mechanisms. Consumption of BE intake reconfigured the gut microbiota composition and reduced the microbial capacity for secondary bile acid metabolism, thereby interrupting bile acid recycling and facilitating fecal excretion. This process led to a reduction in systemic cholesterol levels and ultimately alleviated hepatic lipid accumulation, resulting in enhanced liver health.

## 1. Introduction

As the global economy continues to develop, obesity has surfaced as a significant public health crisis, imposing a considerable burden on healthcare systems worldwide. Currently, more than 2.5 billion adults are overweight, of whom approximately 890 million are classified as obese [1]. Since 1990, the global prevalence rate of adult obesity has more than doubled, while that of adolescent obesity has tripled. This growing epidemic presents a significant threat to public health and the sustainable development of societies, highlighting a critical public health challenge that demands immediate and effective solutions [2,3].

Obesity, commonly acknowledged as a lifestyle-elated metabolic disorder, has been managed via a variety of approved pharmacological treatments, such as orlistat, liraglutide, beinaglutide, and semaglutide [4]. Semaglutide, initially prescribed off-label for obesity treatment, is now approved for weight management in several countries. It is important to note that this drug class can cause adverse reactions, particularly gastrointestinal issues such as nausea, vomiting, and diarrhea [5,6]. Consequently, increasing attention has turned toward dietary-based strategies, and the development of effective functional foods has emerged as a promising approach in combating obesity.

Blueberries (*Vaccinium* spp.), recognized for their substantial polyphenolic compound content, especially anthocyanins, have garnered significant scientific attention. Research findings indicate that the supplementation of blueberries may yield a spectrum of favorable health impacts, possibly mediated via their antioxidant capabilities and other underlying mechanisms. These encompass the delayed progression of neurodegenerative disorders, the preservation of visual capabilities, cardiovascular safeguarding, the augmentation of immune function, and potential anti-cancer efficacy [7,8]. In the context of the global expansion of blueberry cultivation, blueberries are increasingly recognized as promising options for the development of functional foods.

The mammalian gastrointestinal tract hosts a complex and dynamic ecosystem consisting of trillions of commensal microorganisms, which exert crucial functions in nutrient metabolism, absorption, and immune regulation [9]. A growing body of evidence suggests that gut microbiota dysbiosis serves as a causative factor in the pathogenesis of obesity. Key mechanisms encompass enhanced energy harvesting efficiency, perturbed lipid metabolism, compromised intestinal barrier function [10], and chronic low-grade inflammation [11]. Furthermore, gut microbiota act as primary mediators in the metabolism of secondary bile acids. Primary bile acids, which are synthesized from cholesterol in the liver, undergo microbial conversions, such as deconjugation, dehydroxylation, dehydrogenation, and isomerization to the formation of secondary bile acids [12]. These modifications exert a substantial influence on enterohepatic bile acid circulation and regulate host metabolic signaling pathways [13].

Although existing research has predominantly focused on the anti-obesity effects of blueberry supplementation through the regulation of gut microbiota composition and short chain fatty acid (SCFA) production [14,15,16,17], the influence of blueberry extract, especially anthocyanin-rich preparations, on cholesterol metabolism and liver health remains largely uninvestigated. Consequently, this study endeavors to clarify the fundamental mechanisms through which the blueberry extract rich in anthocyanins improves hepatic function by regulating gut microbial activity and its interplay with bile acid metabolism. The results are anticipated to offer novel perspectives for the development of blueberry-derived functional foods aimed at metabolic disorders.

## 2. Materials and Methods

### 2.1. Materials

Blueberry extract rich in anthocyanins (BE) form Shanghai yuanye Bio-Technology Co., Ltd. (Shanghai, China); Alanine aminotransferase (ALT, C009-2-1) Total cholesterol (TC, A111-1-1), Triglyceride (TG, A110-1-1), Low-density lipoprotein cholesterol (LDL-C, A113-1-1), high-density lipoprotein cholesterol (HDL-C, A112-1-1), Glutathione Peroxidase (GSH-px, A005-1-2), Catalase (CAT, A007-1-1), Maleic dialdehyde (MDA, A003-1-2) and Superoxide dismutase (SOD, A001-3-2) from the Biological Research Institute of Nanjing Jiancheng (Nanjing, China), Dihydroethidium (S0063) form Beyotime (Shanghai, China).

### 2.2. Chemical Composition Analysis of Blueberry Extract

A 50 mg sample was dissolved in 500 μL of methanol, shaken for 10 min, and subsequently centrifuged. The supernatant was gathered and filtered through a 0.45 μm filter for subsequent analysis. Through a high performance liquid chromatography system (Vanquish Thermo, Waltham, State MA, USA) coupled with a Hybrid Quadrupole-Orbitrap™ Mass Spectrometer (Q Exactive, Thermo, USA), the chromatographic separation was performed on an ACQUITY UPLC HSS T3 column (100 mm × 2.1 mm, 1.8 μm) with the following gradient program: column temperature at 40 °C; mobile phase A (water containing 0.1% formic acid) and mobile phase B (methanol); flow rate at 0.3 mL/min with a linear gradient setting as follows: 2% B (0–1 min), 2–100% B (1–5.5 min), 100% B (5.5–14 min), 100–2% B (14–14.1 min), 2% B (14.1–16 min).

Mass spectrometry data were acquired within an *m*/*z* range from 150 to 1500, featuring a resolution of 70,000 for MS1 and 17,500 for MS2. The automatic gain control (AGC) target was configured at 1 × 10^6^ for MS1 and 1 × 10^5^ for MS2, with a maximum injection time of 50 ms. The top 10 precursor ions were chosen for tandem mass spectrometry (MS/MS) with normalized collision energy (NCE) settings of 10, 30, and 55. The mass spectrometry files were processed via MS-DIAL 4.70 [18], and the outcomes were searched against the MassBank, Respect, and GNPS databases provided by Dixintai Testing Technology Co., Ltd. (Beijing, China).

### 2.3. Animal Experiment

Twenty-seven C57BL/6 mice (13 females and 14 males, 8 weeks old) was obtained from Shanghai Bikai Laboratory Animal Co., Ltd. (Shanghai, China). The animal experimental protocols were conducted in accordance with the Laboratory Animal Welfare guidelines and were approved by the Animal Ethics Committee of Zhejiang Normal University (Approval number: ZSDW2024075).

Mice were randomly allocated into three groups: a control group, a model group, and a model-treated group. Both the model group and the model-treated group were fed a 60% Kcal high-fat diet (M10160, BIOPIKE, Chengdu, China). The model-treated group received oral gavage of anthocyanin extract every other day (100 mg/kg BE) [15], while the control and model groups were administered an equivalent volume of saline. After 8 weeks of intervention, mice were euthanized under anesthesia. Fresh blood samples were collected and allowed to clot at room temperature for 20 min before being centrifuged to isolate serum. The serum was then stored at −20 °C. cecal contents and liver tissues were also collected, rapidly frozen in liquid nitrogen, and subsequently stored at −80 °C.

### 2.4. Histological and Biochemical Analysis

The concentrations of serum TC, TG, LDL-C, and high-density lipoprotein cholesterol HDL-C were measured in accordance with the kit instructions. The 30 mg liver samples were homogenized with saline at a ratio of 1:9 (*w*/*v*) and subsequently centrifuged at 2500 rpm for 15 min. The supernatant was collected, and the levels of TC, TG, MDA, as well as SOD, CAT, and GSH-px in the liver were measured following the kit instructions. For histopathological evaluation, A single fresh liver lobe was fixed in 4% (*w*/*v*) paraformaldehyde., then dehydrated and processed before being embedded in paraffin. The specimens were sectioned into slices with a thickness of 3 μm and stained using hematoxylin eosin (HE). Additionally, some liver tissues were snap-frozen in liquid nitrogen, cryosectioned into 5-μm-thick sections, and stained with Oil Red O and ROS. All experiments were processed by Yangming Medical Testing (Ningbo, China).

### 2.5. Metabolomics Analysis

Approximately 30 mg of each sample was weighed into a 1.5 mL Eppendorf (EP) tube, followed by the addition of two small steel beads and 400 μL of methanol–water (4:1, *v*/*v*) containing 4 μg/mL of a mixed internal standard. The samples were pre-cooled at −40 °C for 2 min and then homogenized at 45 Hz for 2 min. Subsequently, the samples were sonicated in an ice–water bath for 10 min and left to stand overnight at −40 °C. After incubation, the samples were centrifuged at 12,000 rpm for 20 min at 4 °C, and 150 μL of the supernatant was collected for further analysis.

Quality control (QC) samples were prepared by pooling equal volumes of all extracted samples. Metabolomic analysis was performed using an ACQUITY UPLC I-Class Plus system (Waters, Milford, MA, USA) coupled with a Thermo Q Exactive mass spectrometer (Thermo, USA). Chromatographic separation was achieved using an ACQUITY UPLC HSS T3 column (100 mm × 2.1 mm, 1.8 μm, Waters, USA). The mobile phases consisted of water with 0.1% formic acid (phase A) and acetonitrile (phase B). The gradient elution program was as follows: 0–2 min, 95:5 (A:B, *v*/*v*); 2–4 min, linear change to 70:30; 4–8 min, linear change to 50:50; 8–10 min, linear change to 20:80; 10–14 min, linear change to 0:100; 14–15 min, held at 0:100; 15.1–16 min, re-equilibrated to 95:5. The flow rate was set at 0.35 mL/min, the column temperature at 45 °C, and the injection volume was 2 μL.

Electrospray ionization (ESI) was performed at a temperature of 350 °C, with voltages of +3800 V in positive ion mode and −3000 V in negative ion mode. The ion source gas pressures were set as follows: gas 1 at 55 psi, gas 2 at 60 psi, and curtain gas at 25 psi. The full MS scan was acquired at a resolution of 70,000, and MS/MS scans were acquired at a resolution of 17,500. The normalized collision energy (NCE) was set to stepped values of 10, 20, and 40. All sample processing and metabolomic analysis were outsourced to OE Biotech Co., Ltd. (Shanghai, China).

### 2.6. Proteinomics Analysis

Liver tissues were thoroughly ground in liquid nitrogen and lysed in buffer containing protease inhibitors. The lysates were centrifuged to obtain clear supernatants, and protein concentrations were determined using the BCA assay. Equal amounts of protein were digested overnight with Trypsin-TPCK. The resulting peptides were desalted using a SOLA™ SPE 96-well plate and subsequently dried under vacuum. Dried peptides were reconstituted and separated using a Vanquish Neo UHPLC system (Thermo, USA), followed by data-independent acquisition (DIA) on an Orbitrap Astral mass spectrometer (Thermo, USA) operating in positive-ion mode with a precursor scan range of 380–980 *m*/*z*.

Raw DIA files were analyzed using DIA-NN. Proteins were retained for further analysis if they contained at least one unique peptide, had valid values in at least two samples, and exhibited ≥50% valid-value coverage in at least one experimental group. Data were then median-normalized and log_2_-transformed to obtain reliable protein abundance measurements.

Differentially expressed proteins (DEPs) were identified based on a *p*-value < 0.05 and a fold change > 1.2 or <1/1.2. Identified DEPs were subsequently subjected to Gene Ontology (GO) and Kyoto Encyclopedia of Genes and Genomes (KEGG) pathway enrichment analyses.

### 2.7. Metagenomic Analysis

Metagenomic analysis was conducted to assess the composition and function of the gut microbiota. Microbial community genomic DNA was extracted using the MagPure Soil DNA Kit (MGBio, Guangdong, China), and its concentration and integrity were evaluated. DNA libraries were constructed using the VAHTS Universal Plus DNA Library Prep Kit for Illumina (Vazyme, Nanjing, China). The libraries were sequenced on an Illumina NovaSeq 6000 platform. Subsequent data processing involved cleaning with fastp (v 0.20.1), filtering with bbmap (v 38.93-0), and assembly with MEGAHIT (v 1.2.9) [19,20,21]. ORF prediction was performed using prodigal (v 2.6.3) and translated into amino acid sequences [22]. Gene sets were constructed and aligned using MMSeqs2 (v 13.45111) and Salmon (v 1.8.0). The metagenomic sequencing and analysis were performed by OE Biotech Co., Ltd. (Shanghai, China).

### 2.8. Real-Time qPCR

Total RNA was extracted from liver tissue using RNAiso Plus (Takara, Dalian, China). Approximately 20 mg of tissue was homogenized in 1 mL RNAiso Plus on ice, followed by extraction with 200 μL chloroform for 5 min. After adding one-third volume of absolute ethanol, samples were centrifuged at 12,000× *g* for 15 min. The resulting pellet was washed with 70% ethanol. RNA integrity was verified by 1% agarose gel electrophoresis. Genomic DNA (gDNA) removal and cDNA synthesis were performed using the Hifair^®^ III 1st Strand cDNA Synthesis SuperMix for qPCR (Yeasen, Shanghai, China) according to the manufacturer’s protocol in a T100™ Thermal Cycler (Bio-Rad, Hercules, CA, USA). Quantitative real-time PCR (qPCR) was performed on cDNA from BE-treated mouse livers using Hieff^®^ qPCR SYBR Green Master Mix (Yeasen, Shanghai, China). Relative gene expression was calculated via the 2^−ΔΔCt^ method, with β-actin (Actb) as the reference gene (Table A1).

## 3. Results

### 3.1. Component Analysis

The composition of blueberry anthocyanin extract (BE) was characterized using liquid chromatography–tandem mass spectrometry (LC–MS/MS). Compounds were identified through the matching of precursor ions with reference mass-to-charge (*m*/*z*) values, guaranteeing consistency up to three decimal places. A total of 273 metabolites were annotated using this high-resolution method. Table 1 showcases the 20 most abundant components, encompass bioactive flavonoids such as 3-O-methylquercetin, isorhamnetin, and 2″-O-galloylquercitrin.

Previous research has indicated that these flavonoid compounds manifest a variety of biological activities, such as antioxidant, antimicrobial, and free radical-scavenging properties.

### 3.2. BE Attenuates Body Weight Gain and Ameliorates Lipid Metabolism Disorders in HFD-Fed Mice

**Figure 1:**
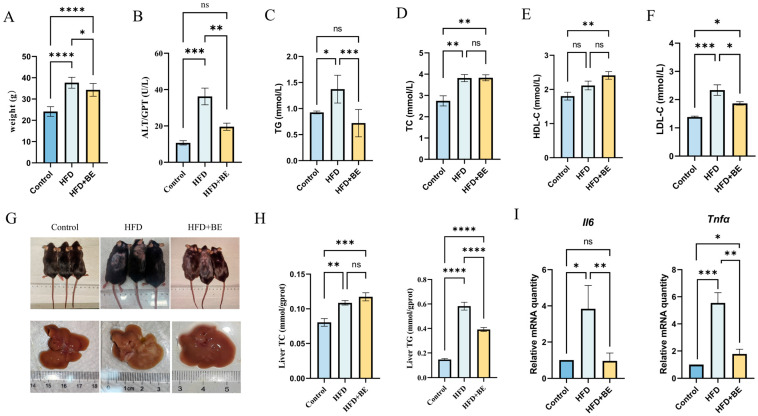
Effects of blueberry anthocyanins on serum parameters and liver in mice. (**A**) Body weight; (**B**) Serum ALT levels; (**C**) Serum TG content; (**D**) Serum TC content; (**E**) Serum HDL-C content; (**F**) Serum LDL-C content; (**G**) Photographs of mice and liver specimens; (**H**) Hepatic TC and TG content; (**I**) Hepatic *Il6* and *Tnfα* mRNA expression levels. Data represent mean ± SEM (n = 6). * *p* < 0.05, ** *p* < 0.01, *** *p* < 0.001, **** *p* < 0.0001.

Body weight of HFD-fed mice rose significantly from 24.16 ± 0.68 g in the Control group to 37.62 ± 0.66 g, confirming successful obesity induction. Following BE intervention, body weight dropped significantly to 34.82 ± 0.87 g, representing a notable reduction compared to the HFD group, though still higher than that of the Control group (Figure 1A).

Morphological assessment indicated that mice fed with a high-fat diet (HFD) displayed characteristic obesity-related alterations, such as dorsal alopecia and pallor of hepatic tissue. Treatment with BE significantly improved the condition of dorsal hair and markedly reduced hepatic lipid accumulation, suggesting a mitigation of hepatic pathology induced by HFD (Figure 1G).

To further evaluate the effects of BE on lipid metabolism, serum levels of total cholesterol (TC), triglycerides (TG), high-density lipoprotein cholesterol (HDL-C), and low-density lipoprotein cholesterol (LDL-C) were assessed. Compared with the Control group, HFD-fed mice showed significant elevations in TC, TG, and LDL-C levels, while HDL-C levels remained unchanged. In contrast, BE supplementation significantly lowered TG levels compared to the HFD group, bringing them close to Control levels. Although TC levels remained elevated, LDL-C levels were significantly decreased in BE-treated mice. Notably, HDL-C levels increased significantly in the BE group compared to both the Control and HFD groups (Figure 1C–F).

Collectively, these findings indicate that HFD feeding induces notable dyslipidemia, and that BE intervention effectively alleviates these lipid metabolic disturbances, particularly by reducing TG and LDL-C levels while enhancing HDL-C.

### 3.3. BE Ameliorates HFD-Induced Hepatic Injury in Mice

HFD feeding significantly elevated serum alanine aminotransferase (ALT) levels (Figure 1B), as well as hepatic total cholesterol (TC) and triglyceride (TG) content. Although BE treatment did not affect hepatic TC levels, it significantly reduced hepatic TG content compared to the HFD group (Figure 1H).

At the molecular level, HFD markedly upregulated hepatic mRNA expression of inflammatory cytokines *Il6* and *Tnfα*. BE administration significantly suppressed the expression of both genes, indicating an anti-inflammatory effect (Figure 1I).

**Figure 2:**
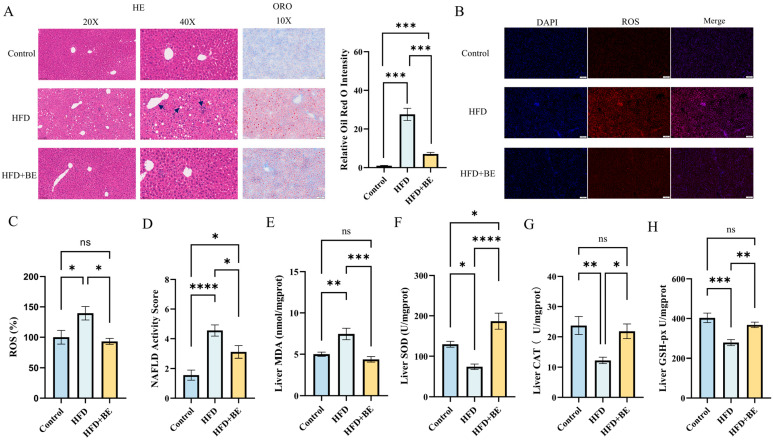
Liver histopathology and oxidative stress markers. (**A**) H&E staining, Oil Red O staining and quantification of Oil Red O in liver sections; (**B**) Hepatic ROS staining; (**C**) ROS quantification; (**D**) NAS scoring; (**E**) Malondialdehyde (MDA) levels; (**F**) Superoxide dismutase (SOD); (**G**) activity; Catalase (CAT) activity; (**H**) Glutathione peroxidase (GSH-Px) activity. Data represent mean ± SEM (n = 6). * *p* < 0.05, ** *p* < 0.01, *** *p* < 0.001, **** *p* < 0.0001.

Histopathological examination via hematoxylin and eosin (HE) staining demonstrated prominent microvesicular steatosis, dispersed inflammatory infiltrates, and disrupted hepatic cord architecture in HFD-fed mice (As shown by the arrow in Figure 2A). Oil Red O staining further confirmed extensive hepatic lipid accumulation. In contrast, BE-treated mice showed notably reduced lipid deposition and attenuated steatotic changes (Figure 2A).

The severity of liver pathology was quantitatively assessed using the NAFLD Activity Score (NAS) system, which evaluates three histological parameters:

(1) Steatosis (0–3 points: 0 ≤ 5%; 1 = 5–33%; 2 = 34–66%; 3 ≥ 66%);
(2) Hepatocellular ballooning (0–2 points: 0 = none; 1 = rare; 2 = frequent);
(3) Lobular inflammation (0–3 points: 0 = none; 1 ≤ 2 foci; 2 = 2–4 foci; 3 ≥ 4 foci);

With a total maximum score of 8. Both HFD and BE groups exhibited significantly elevated NAS values relative to the Control group; however, mice receiving BE showed significantly lower scores than those in the HFD group, indicating amelioration of NAFLD-associated histopathology (Figure 2D).

### 3.4. BE Ameliorate Hepatic Oxidative Stress by Reducing ROS Accumulation and Enhancing Antioxidant Enzyme Activities

Hepatic reactive oxygen species (ROS) staining indicated that, in comparison with the Control group, there was a significantly increased accumulation of ROS in the livers of HFD-fed mice. Conversely, treatment with BE notably decreased the hepatic ROS levels (Figure 2B,C).

Consistent with increased oxidative stress, HFD feeding significantly suppressed the activity of key hepatic antioxidant enzymes, including superoxide dismutase (SOD), catalase (CAT), and glutathione peroxidase (GSH-Px). The BE intervention significantly enhanced the activity of all three enzymes in HFD-fed mice: SOD activity was restored to levels significantly exceeding those of the control group, while CAT and GSH-Px activities were restored to levels comparable to those of the control (Figure 2F–H).

Simultaneously, the hepatic levels of malondialdehyde (MDA), a widely recognized marker of lipid peroxidation, were significantly increased in the HFD group. Furthermore, treatment with BE reduced these levels to values comparable to those of the Control group (Figure 2E).

Collectively, these findings suggest that HFD feeding undermines the hepatic antioxidant defense system, as manifested by the diminished enzymatic activities and elevated levels of ROS and MDA. Treatment with BE significantly reinstated the hepatic antioxidant capacity, implying that the augmentation of enzymatic antioxidant defenses might account for the observed attenuation of oxidative stress.

### 3.5. Proteomic Profiling of Hepatic Tissue Following BE Supplementation

**Figure 3:**
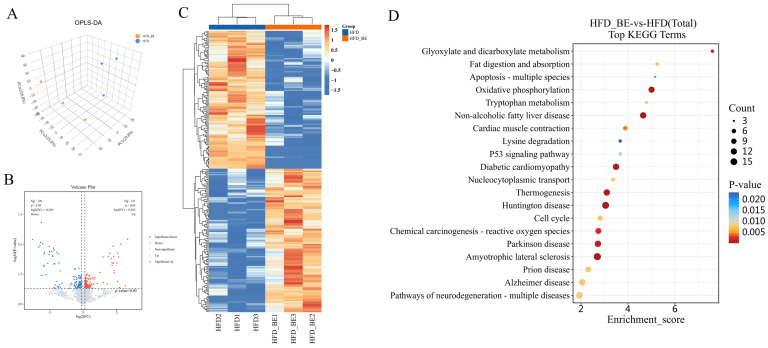
Hepatic proteomic analysis. (**A**) OPLS-DA score plot; (**B**) Volcano plot of differentially expressed proteins; (**C**) Heatmap of differentially expressed proteins; (**D**) KEGG pathway enrichment analysis of differential proteins.

To elucidate the molecular mechanisms by which BE ameliorates HFD-induced hepatic injury, we conducted a proteomic analysis on liver tissues from both the HFD and HFD + BE groups. Orthogonal partial least squares discriminant analysis (OPLS-DA) revealed a clear distinction between the two groups, suggesting distinct proteomic profiles (Figure 3A). After applying a data completeness threshold of ≥50%, 6091 and 6054 proteins were identified in the HFD and HFD + BE groups, respectively.

Using differential expression criteria of *p* < 0.05 and |log_2_(fold change)| ≥ log_2_(1.2), a total of 257 differentially expressed proteins (DEPs) were detected, including 131 upregulated and 126 downregulated proteins in the BE-treated group relative to HFD alone. These DEPs were visualized using volcano plots and heatmaps (Figure 3B,C).

Subsequent Kyoto Encyclopedia of Genes and Genomes (KEGG) pathway enrichment analysis demonstrated significant enrichment in pathways intricately associated with hepatic lipid metabolism and liver injury, particularly Glyoxylate and dicarboxylate metabolism, Oxidative phosphorylation, and Non-alcoholic fatty liver disease (Figure 3D).

### 3.6. Impact of BE on Intestinal Metabolites in HFD-Fed Mice

**Figure 4:**
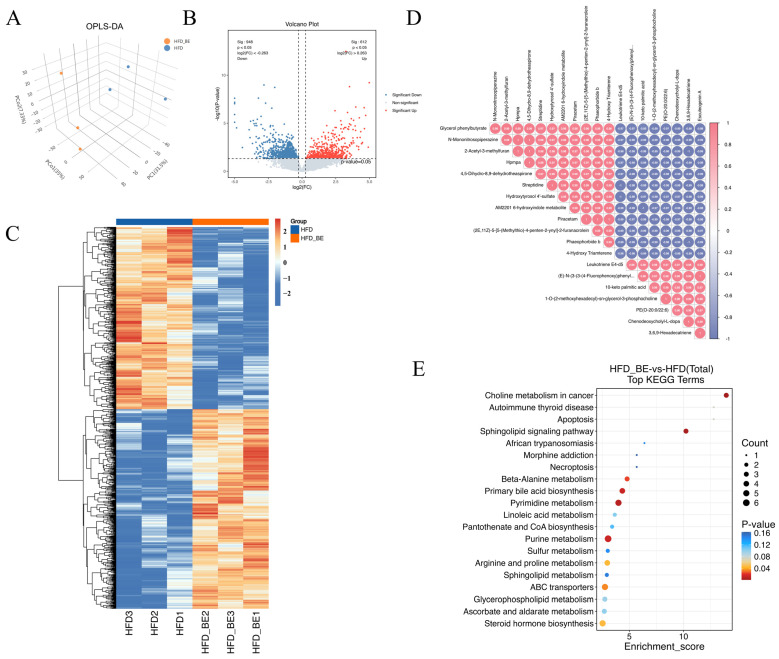
Gut metabolome analysis. (**A**) OPLS-DA score plot; (**B**) Volcano plot of differentially abundant metabolites; (**C**) Heatmap of differentially abundant metabolites; (**D**) Correlation analysis of top 20 DAMs; (**E**) KEGG pathway enrichment analysis of differential metabolites.

To assess the impact of BE intervention on intestinal metabolites, metabolomic profiling was conducted on intestinal contents from HFD and HFD + BE mice. Orthogonal partial least squares discriminant analysis (OPLS-DA) revealed clear separation between the two groups, indicating distinct metabolic profiles (Figure 4A). A total of 8210 metabolites were identified.

Applying differential abundance criteria of *p* < 0.05 and |log_2_(fold change)| ≥ log_2_(1.2), 1004 differentially abundant metabolites (DAMs) were detected in the HFD + BE group compared to the HFD group, including 524 upregulated and 480 downregulated metabolites. These DAMs were visualized by volcano plots and heatmaps (Figure 4B,C). Correlation analysis of the top 20 DAMs revealed notable interrelationships among key differential metabolites (Figure 4D).

KEGG pathway enrichment analysis of DAMs revealed significant enrichment in pathways related to intestinal lipid homeostasis, including primary bile acid biosynthesis, Sphingolipid metabolism, Linoleic acid metabolism, and Steroid hormone biosynthesis (Figure 4E).

**Figure 5:**
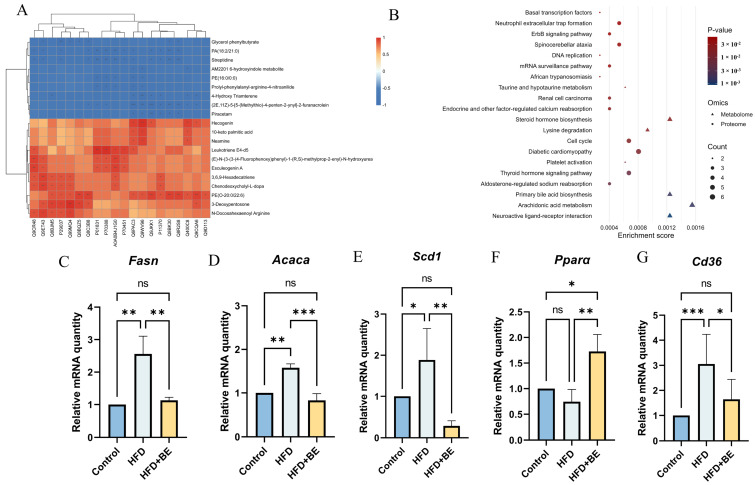
Integrated proteomics-metabolomics analysis. (**A**) Correlation heatmap of top 20 DEPs and DAMs; (**B**) Top 10 enriched KEGG pathways; (**C**) *Fasn* mRNA expression; (**D**) *Acaca* mRNA expression; (**E**) *Cd36* mRNA expression; (**F**) *Scd1* mRNA expression; (**G**) *Pparα* mRNA expression. Data represent mean ± SEM (n = 3). * *p* < 0.05, ** *p* < 0.01, *** *p* < 0.001.

To elucidate the interplay between differentially expressed proteins (DEPs) and differentially abundant metabolites (DAMs) in the HFD + BE group, Spearman correlation analysis was performed on the top 20 DEPs and top 20 DAMs. The resulting correlation heatmap clearly illustrates significant associations between these proteins and metabolites (Figure 5A).

Integrated pathway enrichment analysis combining DEPs and DAMs identified shared pathways. The top 20 significantly enriched pathways, including Primary bile acid biosynthesis, Steroid hormone biosynthesis, Arachidonic acid metabolism, were visualized using a bubble plot (Figure 5B). These pathways are all crucial hepatic lipid metabolism. We paid particular attention to the pathways Primary bile acid biosynthesis, and subsequently conducted targeted validations.

To validate the biological relevance of these multi-omics findings, hepatic mRNA expression of key lipid metabolism-related genes was quantified by real-time PCR. BE supplementation significantly downregulated the aberrantly elevated mRNA levels of *Fasn* (fatty acid synthase), *Acaca* (acetyl-CoA carboxylase alpha), *Cd36* (cluster of differentiation 36), and *Scd1* (stearoyl-CoA desaturase 1) in HFD-fed mice. Conversely, BE markedly upregulated *Pparα* (peroxisome proliferator-activated receptor alpha) expression (Figure 5C–G). These transcriptional changes suggest that BE ameliorates HFD-induced hepatic lipid dysregulation by suppressing fatty acid synthesis (*Fasn*, *Acaca*, *Scd1*) and uptake (*Cd36*), while promoting fatty acid oxidation (*Pparα*).

### 3.7. BE Remodels Gut Microbiota Composition in HFD-Fed Mice

**Figure 6:**
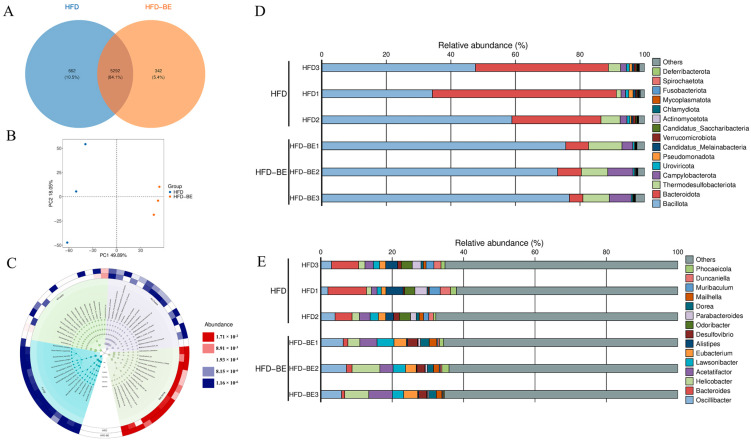
Metagenomic analysis of gut microbiota. (**A**) Venn diagram of species overlap; (**B**) Principal component analysis (PCA) at species level; (**C**) Taxonomic cladogram of top 15 species; (**D**) Phylum-level taxonomic distribution; (**E**) Genus-level taxonomic distribution.

Metagenomic analysis of intestinal contents from HFD and HFD + BE groups was conducted to characterize microbial community profiles. At the species level, 5954 species were identified in HFD mice compared to 5634 species in the HFD + BE group, with 5292 species shared between the two groups. Principal component analysis (PCA) revealed distinct clustering and clear separation between the groups (Figure 6A,B). A taxonomic cladogram illustrated the top 15 most abundant species across taxonomic classifications (Figure 6C).

At the phylum level, BE intervention significantly increased the relative abundances of Bacillota and Thermodesulfobacteriota, while decreasing those of Bacteroidota (Figure 6D). At the genus level, BE supplementation markedly elevated the abundances of *Oscillibacter*, *Helicobacter*, and *Acetatifactor*, while reduced *Bacteroides* (Figure 6E).

**Figure 7:**
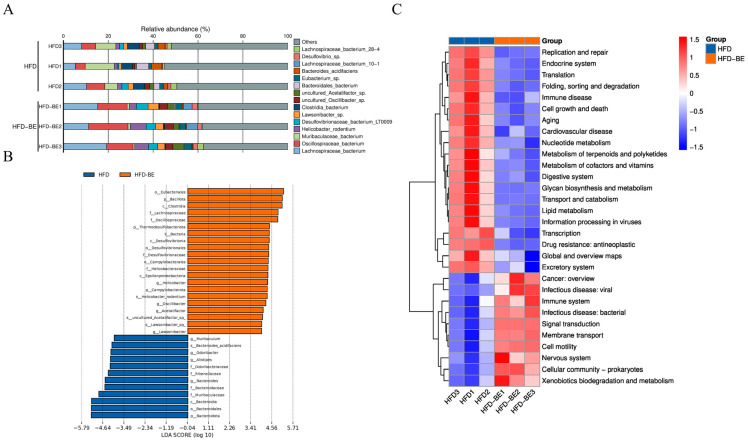
Gut microbiota functional profiling. (**A**) Relative abundance of significantly altered species; (**B**) LEfSe-identified taxa contributing to group differences; (**C**) Hierarchical clustering of top 30 KEGG pathways enriched in HFD + BE group.

Species-level relative abundance data revealed that *Lachnospiraceae bacterium* and *Oscillospiraceae bacterium* were significantly enriched in the HFD + BE group compared to other groups (Figure 7A). Linear discriminant analysis effect size (LEfSe) further identified the taxa that contribute most significantly to intergroup differences (Figure 7B).

Functional annotation of gut microbial genes via the Kyoto Encyclopedia of Genes and Genomes (KEGG) database revealed the top 30 enriched pathways, visualized by hierarchical clustering heatmap (Figure 7C). Pathways related to lipid metabolism and cardiovascular disease were notably enriched, indicating that changes in microbial composition induced by BE may affect the host’s lipid metabolic processes.

**Figure 8:**
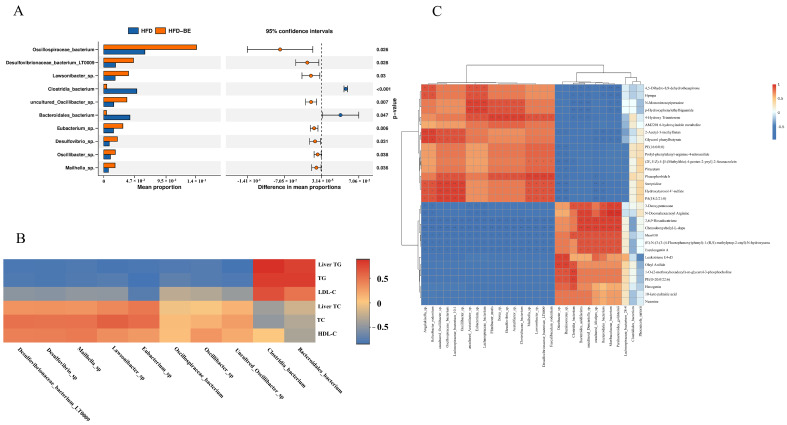
Microbiota-host correlation networks. (**A**) Top 10 differentially abundant species at species level; (**B**) Spearman correlations between differential gut species and serum/hepatic biomarkers; (**C**) Correlation heatmap of top 30 abundant species and intestinal metabolites. * *p* < 0.05, ** *p* < 0.01.

To elucidate microbiota-host interactions, we profiled the top 10 species that exhibited significant alterations at the species level. Health-associated taxa, including *Oscillibacter* sp., *Oscillospiraceae* bacterium, and *Eubacterium* sp., were markedly enriched in the BE-treated group. (Figure 8A). Spearman correlation analysis further demonstrated significant correlations between these differentially abundant gut species and crucial serum and hepatic biochemical parameters (Figure 8B).

Furthermore, Spearman correlation was applied to analyze the relationships between the top 30 abundant gut species and intestinal metabolites. The resulting heatmap illustrates species-metabolite associations, indicating potential crosstalk between the gut microbiota and host metabolic outputs (Figure 8C).

### 3.8. BE Modulate Bile Acid Metabolism in HFD-Fed Mice

**Figure 9:**
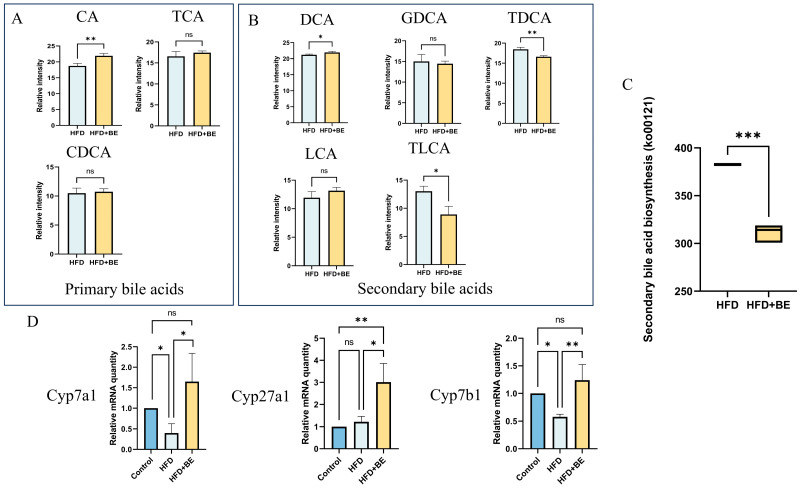
Bile acid metabolism remodeling by BE. (**A**) Primary bile acids: CA, CDCA, TCA; (**B**) Secondary bile acids: LCA, DCA, TLCA, GDCA, TDCA; (**C**) KEGG functional prediction of gut microbiota (ko00121: secondary bile acid biosynthesis); (**D**) Hepatic mRNA expression of bile acid synthesis rate-limiting enzymes *Cyp7a1*, *Cyp27a1*, and *Cyp7b1*. * *p* < 0.05, ** *p* < 0.01, *** *p* < 0.001.

An integrated analysis of intestinal metabolomics and hepatic proteomics revealed significant co-enrichment in pathways related to bile secretion and cholesterol metabolism (KEGG), indicating that BE intervention substantially remodels bile acid (BA) dynamics in HFD-fed mice. Specifically, compared to HFD controls, the HFD + BE group exhibited significantly increased intestinal levels of cholic acid (CA), whereas chenodeoxycholic acid (CDCA) and taurocholic acid (TCA) remained unchanged (Figure 9A).

Secondary bile acids exhibited heterogeneous changes: deoxycholic acid (DCA) was significantly increased, whereas taurodeoxycholic acid (TDCA) and taurolithocholic acid (TLCA) were markedly decreased; by contrast, glycodeoxycholic acid (GDCA) and lithocholic acid (LCA) showed no significant alteration. These shifts align with metagenomic functional predictions indicating inhibition of the secondary bile acid biosynthesis pathway (Figure 9B,C).

Concomitantly, BE significantly upregulated hepatic mRNA expression of rate-limiting enzymes involved in BA synthesis, including *Cyp7a1*, *Cyp27a1*, and *Cyp7b1*, suggesting enhanced hepatic BA synthesis that facilitates cholesterol catabolism and mitigates hepatic cholesterol accumulation(Figure 9D).

Notably, the reduction in intestinal TLCA and TDCA may contribute to the attenuation of oxidative stress by suppressing lipid peroxidation, thereby reducing hepatic malondialdehyde (MDA) levels. Although primary bile acids are typically converted to secondary bile acids by gut bacteria, BE-induced restructuring of the gut microbiota in HFD-fed mice appears to impede this biotransformation, resulting in primary BA retention. This retention likely activates hepatic farnesoid X receptor (FXR), leading to suppression of lipogenic gene expression (e.g., *Fasn*, *Acaca*) and subsequent amelioration of hepatic lipid accumulation.

## 4. Discussion

Modern lifestyles often lead to a consistent intake of calories that surpasses metabolic needs, resulting in excessive fat accumulation that contributes to the development of metabolic syndrome, type 2 diabetes mellitus, and hypertension [28,29,30]. As a major contributor to obesity, dietary interventions can effectively alleviate the health risks associated with obesity. In this research, mice fed with a high-fat diet (HFD) demonstrated notable weight gain, dyslipidemia, and hepatic pathological changes, including hepatocellular ballooning and vacuolar degeneration. Intervention with blueberry-derived BE significantly decreased serum lipid levels and hepatic lipid content, concurrently alleviating liver injury. This hepatoprotective effect was mechanistically attributed to BE-enhanced antioxidant enzyme activity and a reduction in reactive oxygen species (ROS) accumulation, consistent with established evidence that blueberry anthocyanins alleviate obesity and lipid deposition induced by HFD [14,15,16].

Notably, this study, for the first time, systematically elucidates the direct regulatory effects of BE on liver health. Specifically, BE coordinately suppresses hepatic lipogenesis by downregulating *Fasn* (fatty acid synthase), *Acaca* (acetyl-CoA carboxylase), and *Scd1* (stearoyl-CoA desaturase 1), while simultaneously inhibiting *Cd36* mediated (cluster of differentiation 36) exogenous lipid uptake. Importantly, BE also activates *Pparα* (peroxisome proliferator-activated receptor alpha), thereby promoting fatty acid β-oxidation. In line with our findings, previous studies have demonstrated that the anthocyanin cyanidin-3-O-glucoside effectively alleviates hepatic lipid accumulation and improves systemic glucose metabolism [31].

At the gut microbial level, BE significantly enriched beneficial taxa, including *Oscillibacter* sp. and uncultured *Oscillibacter* species. Consistent with our findings, recent work demonstrated that individual anthocyanins such as M3G and M35G exert distinct modulatory effects on gut microbiota composition in HFD-fed mice, highlighting both the shared and structure-specific actions of anthocyanins in shaping host–microbe interactions [32]. Considering *Oscillibacter*’s established role in cholesterol catabolism and its inverse correlation with serum cholesterol levels, this shift likely contributes to enhanced lipid metabolism [33]. Notably, this shift intersects with bile acid (BA) metabolic reprogramming. Integrated multi-omics analysis revealed that BE intervention induced several characteristic changes: it elevated the level of the primary bile acid cholic acid (CA) without significantly affecting chenodeoxycholic acid (CDCA) or taurocholic acid (TCA); it suppressed the secondary bile acid biosynthesis pathway (ko00121); and it led to divergent alterations in secondary bile acids, including increased deoxycholic acid (DCA), decreased taurodeoxycholic acid (TDCA) and taurolithocholic acid (TLCA), while glycodeoxycholic acid (GDCA) and lithocholic acid (LCA) remained unchanged.

Previous studies have demonstrated that anthocyanins from Butterfly Pea Flower promote hepatic bile acid synthesis and elevate intestinal primary bile acid levels, thereby activating FXR [34]. Consistent with these findings, our study also observed dual-pathway activation of hepatic bile acid synthesis, as reflected by the upregulation of the rate-limiting enzyme *Cyp7a1* in the classical pathway and *Cyp27a1* in the alternative pathway. The resulting accumulation of primary BAs is presumed to activate hepatic FXR, which subsequently represses lipogenic gene expression, establishing a core regulatory axis within the gut–liver network. Meanwhile, increased fecal bile acid excretion may enhance hepatic cholesterol clearance, reduce hepatic cholesterol accumulation, and help maintain bile acid homeostasis, ultimately alleviating hepatic steatosis.

Although our multi-omics analysis provides the initial integrative evidence that blueberry-derived anthocyanins alleviate HFD-induced hepatic injury in mice, it is imperative to recognize several limitations. Firstly, the extract utilized in this study also comprised non-anthocyanin metabolites, which hindered our ability to attribute the observed benefits solely to anthocyanins and suggested the potential for synergistic effects from other blueberry components. Secondly, our metabolomic profiling was confined to the gut compartment; without a parallel hepatic metabolome analysis, the connections between anthocyanin intake, decreased ROS levels, and remodeled lipid metabolism could only be inferred indirectly through histological and biochemical data. Thirdly, we did not ascertain a direct causal relationship between the changes in gut microbiota induced by anthocyanins and bile acid metabolism, and the progression of hepatic steatosis. Future studies will therefore employ chromatographic isolation and high-resolution mass spectrometry to obtain purified anthocyanin species, alongside comprehensive liver metabolomics, to delineate the molecular mechanisms underlying their hepatoprotective effects. Such efforts will ultimately support the rational development of blueberry-based functional foods.

## Figures and Tables

**Table 1:** TOP 20 composition of Blueberry anthocyanins Extract.

Components	RT (min)	Precursor *m*/*z*	Reference *m*/*z*	Error (ppm)	Adduct	Formula	Ontology
JACAGLABROSIDE B	6.697467	621.10520	621.10486	0.547412	[M+H]^+^	C_30_H_30_O_13_	Saccharolipids
3-O-methylquercetin [23]	7.154383	317.06520	317.06500	0.630785	[M+H]^+^	C_16_H_12_O_7_	3-O-methylated flavonoids
Isorhamnetin [24]	8.0744	317.06540	317.06558	−0.567706	[M+H]^+^	C_16_H_12_O_7_	Flavonols
h_39_boldenone	9.193566	269.18930	269.18900	1.114459	[M+H]^+^	C_19_H_26_O_2_	Androgens and derivatives
Flavanone base + 6O	7.242517	319.04570	319.04590	−0.62687	[M-H]^−^	C_15_H_12_O_8_	Epigallocatechins
Glucose-6-phosphate	7.767416	259.02470	259.02469	0.038606	[M-H]^−^	C_6_H_13_O_9_P	Hexose phosphates
2″-O-Galloylquercitrin	6.697467	623.10280	623.10260	0.320974	[M+H]^+^	C_28_H_24_O_15_	Flavonoid-3-O-glycosides
Melophlin B/C/G/K/L/N/O	7.37585	324.25320	324.25299	0.647642	[M+H]^+^	C_19_H_33_NO_3_	N-alkylpyrrolidines
Kaempferol 3-O-rhamninoside [25]	6.931483	779.18090	779.18066	0.308016	[M+H]^+^	C_33_H_40_O_19_	Flavonoid-3-O-glycosides
4′-O-(2′-E-Coumaroyl GluA)(1-2)GluA) Apigenin	6.84615	767.14640	767.14600	0.521413	[M-H]^−^	C_36_H_32_O_19_	Flavonoid O-glucuronides
(2s,3s)-3,5,7-trihydroxy-6-methyl-2-(3,4,5-trihydroxyphenyl)chroman-4-one	7.53415	333.06150	333.06158	−0.2402	[M-H]^−^	C_16_H_14_O_8_	Epigallocatechins
Triptophenolide [26]	9.193566	313.17920	313.17911	0.287375	[M+H]^+^	C_20_H_24_O_3_	Oxosteroids
2-Methoxyestradiol [27]	8.739667	303.19510	303.19501	0.296839	[M+H]^+^	C_19_H_26_O_3_	Estrogens and derivatives
Haemoventosine	6.4543	305.06410	305.06400	0.3278	[M+H]^+^	C_15_H_12_O_7_	Isochromanequinones
1′,2′-dihydro-2′,6-dihydroxyrotenone	7.68385	451.19580	451.19574	0.13298	[M+H]^+^	C_23_H_24_O_8_	Rotenones
(9Z,12E)-15,16-dihydroxyoctadeca-9,12-dienoic acid	8.03525	313.23710	313.23734	−0.76619	[M+H]^+^	C_18_H_32_O_4_	Lineolic acids and derivatives
Beta-Peltatin	8.230583	413.12410	413.12418	−0.19365	[M-H]^−^	C_22_H_22_O_8_	Lignan lactones
3-HYDROXY-4-(SUCCIN-2-YL)-CARYOLANE delta-LACTONE	8.18775	321.20570	321.20596	−0.80945	[M+H]^+^	C_19_H_28_O_4_	Delta valerolactones
(-)-Bilobalide	7.280933	325.09270	325.09290	−0.61521	[M-H]^−^	C_15_H_18_O_8_	Ginkgolides and bilobalides
Linoleoyl Ethanolamide	8.36255	324.28930	324.28900	0.925101	[M+H]^+^	C_20_H_37_NO_2_	N-acylethanolamines

## Data Availability

The raw sequence data have been submitted to the NCBI Short Read Archive (SRA) with accession number <PRJNA1288690>. The metabolomics raw data have been uploaded to the MetaboLights project number <MTBLS12701>. The raw proteomics data have been deposited in the OMIX, China National Center for Bioinformation/Beijing Institute of Genomics, Chinese Academy of Sciences <OMIX010945>. The original contributions presented in the study are included in the article; further inquiries can be directed to the corresponding authors.

## Appendix A

**Table A1:** Primer.

Gene Symbol	Forward Primer	Reverse Primer
Actb	GGCTGTATTCCCCTCCATCG	CCAGTTGGTAACAATGCCATGT
Il6	CTGCAAGAGACTTCCATCCAG	AGTGGTATAGACAGGTCTGTTGG
Tnf F	CAGGCGGTGCCTATGTCTC	CGATCACCCCGAAGTTCAGTAG
Cd36	ATGGGCTGTGATCGGAACTG	TTTGCCACGTCATCTGGGTTT
Fasn	GGAGGTGGTGATAGCCGGTAT	TGGGTAATCCATAGAGCCCAG
Scd1	TTCTTGCGATACACTCTGGTGC	CGGGATTGAATGTTCTTGTCGT
Ppara	AGAGCCCCATCTGTCCTCTC	ACTGGTAGTCTGCAAAACCAAA
Acaca	GATGAACCATCTCCGTTGGC	GACCCAATTATGAATCGGGAGTG
Cyp7a1	ACACTACTTCTGCGAAGGCAT	CCCGGAGGTCTCATGACAGA
Cyp27a1	CCAGGCACAGGAGAGTACG	GGGCAAGTGCAGCACATAG
Cyp7b1	GGAGCCACGACCCTAGATG	TGCCAAGATAAGGAAGCCAAC
